# A Ciliary Protein EVC2/LIMBIN Plays a Critical Role in the Skull Base for Mid-Facial Development

**DOI:** 10.3389/fphys.2018.01484

**Published:** 2018-10-25

**Authors:** Anshul K. Kulkarni, Ke’ale W. Louie, Marilia Yatabe, Antonio Carlos de Oliveira Ruellas, Yoshiyuki Mochida, Lucia H. S. Cevidanes, Yuji Mishina, Honghao Zhang

**Affiliations:** ^1^Department of Biologic and Materials Sciences, School of Dentistry, University of Michigan, Ann Arbor, MI, United States; ^2^Department of Orthodontics and Pediatric Dentistry, School of Dentistry, University of Michigan, Ann Arbor, MI, United States; ^3^Department of Molecular and Cell Biology, Henry M. Goldman School of Dental Medicine, Boston University, Boston, MA, United States

**Keywords:** EvC syndrome, Evc2, Limbin, ciliopathy, mid-facial defects, skull base, neural crest

## Abstract

Ellis-van Creveld (EvC) syndrome is an autosomal recessive chondrodysplastic disorder. Affected patients present a wide spectrum of symptoms including short stature, postaxial polydactyly, and dental abnormalities. We previously disrupted *Evc2*, one of the causative genes for EvC syndrome, in mice using a neural crest-specific, *Cre*-mediated approach (i.e., P0-*Cre*, referred to as *Evc2 P0* mutants). Despite the fact that *P0-Cre* predominantly targets the mid-facial region, we reported that many mid-facial defects identified in *Evc2* global mutants are not present in *Evc2 P0* mutants at postnatal day 8 (P8). In the current study, we used multiple Cre lines (*P0-Cre* and *Wnt1-Cre*, respectively), to specifically delete *Evc2* in neural crest-derived tissues and compared the resulting mid-facial defects at multiple time points (P8 and P28, respectively). While both Cre lines indistinguishably targeted the mid-facial region, they differentially targeted the anterior portion of the skull base. By comprehensively analyzing the shapes of conditional mutant skulls, we detected differentially affected mid-facial defects in *Evc2 P0* mutants and *Evc2 Wnt1* mutants. Micro-CT analysis of the skull base further revealed that the *Evc2* mutation leads to a differentially affected skull base, caused by premature closure of the intersphenoid synchondrosis (presphenoidal synchondrosis), which limited the elongation of the anterior skull base during the postnatal development of the skull. Given the importance of the skull base in mid-facial bone development, our results suggest that loss of function of *Evc2* within the skull base secondarily leads to many aspects of the mid-facial defects developed by the EvC syndrome.

## Introduction

Ellis-van Creveld (EvC) syndrome is an autosomal recessive chondroectodermal dysplasia (McKusick et al., 1964). Affected individuals display a wide spectrum of symptoms including dwarfism, postaxial polydactyly, nail dysplasia, atrial septal or atrioventricular septal cardiovascular defects, and dental anomalies (McKusick et al., 1964; Baujat and Le Merrer, 2007). Genetic studies have allowed identification of two causative genes for EvC syndrome, *EVC* and *EVC2*, and homozygous mutations in either have been linked to two-thirds of EvC patients (Ruiz-Perez et al., 2003). Additionally, EvC syndrome has been characterized as a ciliopathy because the EVC and EVC2 proteins are intracellularly localized at the bottom of the primary cilium, where they form a heterotrimeric protein complex with SMO and transduce Hedgehog signaling (Dorn et al., 2012; Caparros-Martin et al., 2013). Our previous studies interestingly identified the bovine ortholog *EVC2/LIMBIN* as a causative gene for chondrodysplasia in Japanese brown cattle (Takeda et al., 2002). Recent studies also identify *EVC2/LIMBIN* mutations in dwarf Tyrolean Gray cattle (Murgiano et al., 2014). These two studies suggest an evolutionarily conserved function of *EVC2* among different species.

Multiple studies have linked EvC syndrome with a wide range of craniofacial abnormalities including an enlarged skull, depressed nasal bridge, class II skeletal pattern with or without mandibular prognathism, and a skeletal open bite. Patients less frequently exhibit normocephaly with minimal facial defects and instances of class I skeletal patterns (Ellis and van Creveld, 1940; Eidelman et al., 1965; Prabhu et al., 1978; da Silva et al., 1980; Varela and Ramos, 1996). The variation of mid-facial defects reported in EvC patients prompted the generation of mouse models for EvC syndrome that facilitate characterization of affected mid-facial development and associated pathological mechanisms.

We and others have used both *Evc* and *Evc2* mutant mice to characterize different aspects of abnormal development (Ruiz-Perez et al., 2007; Caparros-Martin et al., 2013; Zhang et al., 2015). Generating our own *Evc2* mutant mice enabled us to characterize the pathological mechanisms leading to abnormal appendicular bone and hypomorphic enamel development (Zhang et al., 2015, 2016a,b). Our subsequent studies further demonstrated the expression of *Evc2* in the mid-facial regions (Badri et al., 2016a) and mid-facial defects in *Evc2* global mutant mice (Badri et al., 2016b). We most recently employed a conditional, *Cre*-mediated approach toward deleting *Evc2* in a neural crest-specific manner. In a comparison between global and *P0-Cre-*mediated *Evc2* mutants, we reported that at postnatal day 8 (P8): (1) *Evc2 P0* mutants somewhat recapitulate craniofacial and tooth phenotypes exhibited by *Evc2* global mutant mice and that (2) many mid-facial defects identified in the *Evc2* global mutants are not present in the *Evc2 P0* mutants (Kwon et al., 2018). This latter finding was surprising and warranted further study since *P0-Cre* predominantly targets the mid-facial region where *Evc2* is, similarly, expressed.

To identify a pathological mechanism leading to the mid-facial defects in *Evc2* mutant mice and EvC patients, we compared outcomes of *Evc2* deletion within the neural crest-derived tissues of two similar, but not identical, neural crest-specific *Cre* lines: *P0-Cre* and *Wnt1-Cre*. Recombination efficiency and craniofacial deficiencies varied between mouse lines and facial region. Specifically, although both *Cre* lines showed indistinguishable recombination efficiency within the mid-facial bones, *Wnt1-Cre*-mediated excision of *Evc2* resulted in a more robust mid-facial hypoplasia. Conversely, those two Cre lines showed differential recombination efficiencies within the anterior portion of the skull base. Analyses of these two mutant mice lines highlight the critical function of the skull base during postnatal mid-facial development and uncovers the pathological mechanisms whereby the shortened skull base, due to *Evc2* loss of function within those bones, leads to characteristic EvC mid-facial defects.

## Materials and Methods

### Animal Model

Animals were maintained and used in compliance with the Institutional Animal Care and Use Committee (IACUC) of the University of Michigan in accordance with the National Institutes of Health Guidelines for Care and Use of Animals in research, and all experimental procedures were approved by the IACUC of the University of Michigan. *Evc2* floxed mice used in this study were generated by our group and reported previously (Zhang et al., 2015). Neural crest-specific *Evc2* mutant mice were generated by crossing *Evc2* floxed mice either with *Wnt1-Cre* mice (Danielian et al., 1998) or with *P0*-Cre mice, *C57BL/6J-Tg(P0-Cre)94Imeg* (ID 148), which was provided by Dr. Kenichi Yamamura (Yamauchi et al., 1999). All mice were maintained in a mixed background of C57BL6/J and 129S6 and were crossed and maintained in our semi-closed mouse colony for at least 5 years.

### Micro-CT (μCT)

Micro-CT scanning of fixed heads was performed at the University of Michigan using a Micro-CT Core (μCT100 Scanco Medical, Bassersdorf, Switzerland). Scan settings were as following: voxel size 12 μm, 55 kVp, 109 μA, 0.5 mm AL filter, and integration time 500 ms.

### Cephalometric Analysis

Lateral radiographic films were taken according to previously published methods. The linear and angular cephalometric analyses followed the parameters outlined in a previous report (Engstrom et al., 1982) with certain modifications (Table 1). An additional landmark (Br) was placed on the bregma, the point of intersection between the sagittal and coronal sutures (see Supplementary Table S1). The distance from the bregma (Br) to the most posterior point of the skull (Po) was measured, denoted by the abbreviation Po-Br (see Supplementary Table S1). The angle ABrL/PoBrL was also measured between the Po-Br segment described above and the A-Br segment, which stretches the combined length of the nasal and frontal bones. This angle was used to emulate the concavity of the skull superficial to the cranial vault. Overall, 15 landmarks (Table 1) were placed in each sample and a total of 20 linear measurements and 15 angular measurements were taken (Table 1).

**Table 1:** Description of landmarks and landmark-associated linear and angular measurements.

Landmark	Description		
A	The most anterior point on the nasal bone.		
Bl	The intersection between the lingual surface of the lower incisors and the most anterior part of the lingual alveolar bone.		
Br	The bregma, the point of intersection between the sagittal and coronal sutures.		
Bu	A point on the premaxilla between jaw bone and the lingual surface of the upper lingual incisors.		
E	The intersection between the frontal bone and the most superior-anterior point of the posterior limit of the ethmoid bone.		
Id	The most inferior and anterior point on the alveolar process of the mandible.		
Ii	The most prominent point between the incisal edges of the lower incisors.		
Iu	The most prominent point between the incisal edges of the upper incisors.		
Ml	The intersection between the mandibular alveolar bone and the mesial surface on the first molar.		
Mn	A point in the deepest part of the antegonial notch curvature.		
Mu	The intersection between the maxillary bone and the mesial surface of the upper first molar.		
N	A point on the nasofrontal suture.		
Po	The most posterior point on the cranial vault.		
Pr	The most inferior and anterior point on the alveolar process of the premaxilla.		
So	The intersection between the posterior border of the basisphenoid and the tympanic bulla.		

**Landmarks**	**Segment**	**Landmarks**	**Angle**

A-N	Nasal bone length	PoEL/SoEL	Cranial vault to cranial base
A-Pr	Nasal bone height	ANL/SoEL	Nasal bone to cranial base
E-Mu	Viscerocranial height	ANL/PoEL	Nasal bone to cranial vault
E-Iu	Growth axis of upper face	ANL/PrNL	Nasal bone to premaxilla
E-Bu	Viscerocranial length (posterior to anterior incisors)	MuBuL/SoEL	Maxilla-premaxilla to cranial base
E-Pr	Viscerocranial length (anterior to anterior incisors)	MuBuL/PoEL	Maxilla-premaxilla to cranial vault
N-Pr	Relative position of pre-maxilla to cranium	PrEL/SoEL	Premaxilla to cranial base
Mu-Pr	Distance between molar and incisor in maxilla	BuEL/PoEL	Premaxilla to cranial vault
Mu-Bu	Palatal length	BuEL/SoEL	Upper incisors to cranial base
Mn-Id	Mandibular corpus length	IuEL/PoEL	Upper incisors to cranial vault
Ml-Bl	Mandibular lingual alveolar bone length	MuBuL/PrluL	Upper incisor inclination
Pr-Iu	Erupted upper incisor length	MlliL/ldLiL	Lower incisor inclination
Id-Ii	Erupted lower incisor length	ABrL/PoBrL	Angle of cranial vault
Ml-Ii	Distance between molar and incisor in mandible	PrEL/PoEL	Premaxilla to cranial vault
So-E	Length of anterior cranial base		
Po-E	Neurocranial length		
Po-Br	Length of posterior cranium		
Po-Mu	The distance between the first molar to the most posterior point		
Po-A	Total skull length		

### Image Acquisition, Segmentation, and Surface Model Analysis

We performed shape comparisons of the nasal, frontal and parietal bones between controls and mutants (*Evc2^fl/fl^*; *Wnt1-Cre*). The surface model for each bone was generated based on the micro-CT data, using ITK-SNAP (open-source software developed by grants and contracts from the United States National Institutes of Health^1^). For shape comparisons, landmarks were placed on the individual bone surfaces by using “modules” developed in 3D Slicer (open-source software ^2^). 3D Slicer modules were then used to both superimpose the two bones based on the landmarks (CMF Registration) and to visualize shape differences (Shape Population Viewer). Model superimpositions for the mid-line region of the skulls were carried out using 3D Slicer with the following landmarks on the interparietal bones and basioccipital bones: right and left anterolateral tips of the interparietal bone, cross point between the median line and the line which connects left and right anterolateral tip of the interparietal bone, and posterior tip of the interparietal bone. Model superimpositions for nasal, frontal and parietal bones were carried out using 3D Slicer with landmarks on the right and left of anterolateral and posterior tips of each bone. Superimpositions were achieved by posterior registration of the two models. The length of the skull base and each indicated segment of the skull base were determined by assessing the linear distance between the most anterior midline point of and the most posterior point on the midline region of the skull base or the indicated segment of the skull base using a module program, Q3DC, in 3D Slicer.

### Statistical Analysis

The Mann–Whitney *U* test was done by using SPSS21.0 to evaluate the linear and angular measurements between controls and mutants.

## Results

### *Evc2* Mutation Within Neural Crest Cells Leads to Craniofacial Abnormalities

**FIGURE 1:**
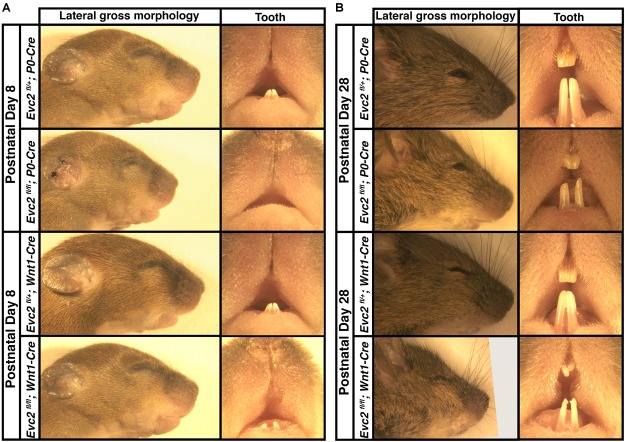
Gross morphologies of heads from *Evc2 P0* mutants and *Evc2 Wnt1* mutants at P8 **(A)** and P28 **(B)**.

Our previous studies indicated that global *Evc2* mutation led to retarded growth and significantly affected both body and head size (Zhang et al., 2015; Badri et al., 2016b). To better characterize the function of *Evc2* in craniofacial development, we generated mutant mice with *Evc2* deleted in the neural crest-derived cells. Both *P0-Cre* and *Wnt1-Cre* mediate nearly identical recombination efficiencies within the mid-facial region (Danielian et al., 1998; Yamauchi et al., 1999) (our own comparisons, data not shown). However, *Evc2* deletion mediated by the *P0-Cre* (*Evc2^fl/fl^*; *P0-Cre*, abbreviated as *Evc2 P0* mutants) leads to an overall shortened head and delayed mandibular incisor eruption at postnatal day 8 (P8) (Figure 1A). In *Evc2 P0* mutants at P28, we observed less pronounced reductions in head and incisor lengths (Figure 1B). *Evc2* deletions mediated by *Wnt1-Cre* (*Evc2^fl/fl^*; *Wnt1-Cre*, abbreviated as *Evc2 Wnt1* mutants) did not affect head length at P8 but did lead to shortened head and hypomorphic incisor dimensions at P28 (Figures 1A,B). Unlike *Evc2* global mutant mice, there was no apparent neonatal or postnatal death observed in either *Evc2 P0* or *Evc2 Wnt1* mutant groups at the above time points. Furthermore, in both types of *Evc2* cKO, at P8 and P28, we observed insignificant gender-specific differences (Supplementary Table S1), particularly in the parameters characterizing mid-facial defects. Lack of gender-specific observations is consistent with previous studies and we, therefore, did not match genders in our comparative analyses (Vora et al., 2015; Wei et al., 2017). Therefore, we combined both genders for the rest of our comparisons.

### *Evc2 P0* Mutants Showed More Aspects of Mid-Facial Defects Than *Evc2 Wnt1* Mutants at P8

**FIGURE 2:**
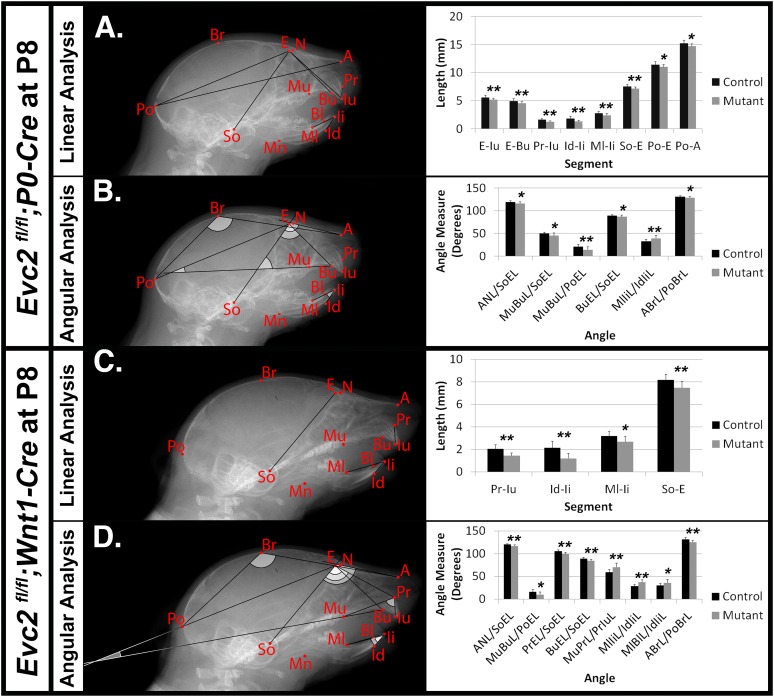
Differential mid-facial defects between *Evc2 P0* mutants and *Evc2 Wnt1* mutants at P8. Lateral X-ray cephalogram indicates the linear **(A)** and angular **(B)** measurements with significant differences between controls and *Evc2 P0* mutants. Lateral X-ray cephalogram indicates the linear **(C)** and angular **(D)** measurements with significant differences between controls and *Evc2 Wnt1* mutants. Graphs indicate the linear and angular values of the measurement with significant differences (*N* = 7; ^∗^*p* < 0.05; and ^∗∗^*p* < 0.01).

To characterize the abnormal craniofacial development observed in both *Evc2 P0* mutants and *Evc2 Wnt1* mutants, we examined the linear and angular dimensions from lateral cephalometric radiographs. In *Evc2 P0* mutants at P8, the lateral cephalometric analysis indicated abnormalities in the incisors, skull base, and mid-facial regions. We specifically found decreases in growth axes of the upper face (E-Iu), viscerocranial length (E-Bu), erupted upper incisor length (Pr-Iu), erupted lower incisor length (Id-Ii), distance between the first molar and incisor in mandible (Ml-Ii), length of anterior cranial base (So-E), neurocranial length (Po-E), the distance between the first molar to the most posterior point (Po-Mu), and total skull length (Po-A), respectively (Figure 2A and Table 2). These phenotypic observations recapitulate many of the mid-facial defects of the *Evc2* global mutants that we previously reported (Badri et al., 2016a). Additionally, we found a shallow mid-facial region in the *Evc2 P0* mutants, as evidenced by decreased angle measures in nasal bone to cranial base (ANL/SoEL), maxilla-premaxilla to cranial base (MuBuL/SoEL), maxilla-premaxilla to cranial vault (MuBuL/PoEL), upper incisors to cranial base (BuEL/SoEL), and angle of cranial vault (ABrL/PoBrL), respectively (Figure 2B and Table 2).

**Table 2:** Summary of phenotypic spectrums of *Evc2* global mutants, *P0* mutants and *Wnt1* mutants.

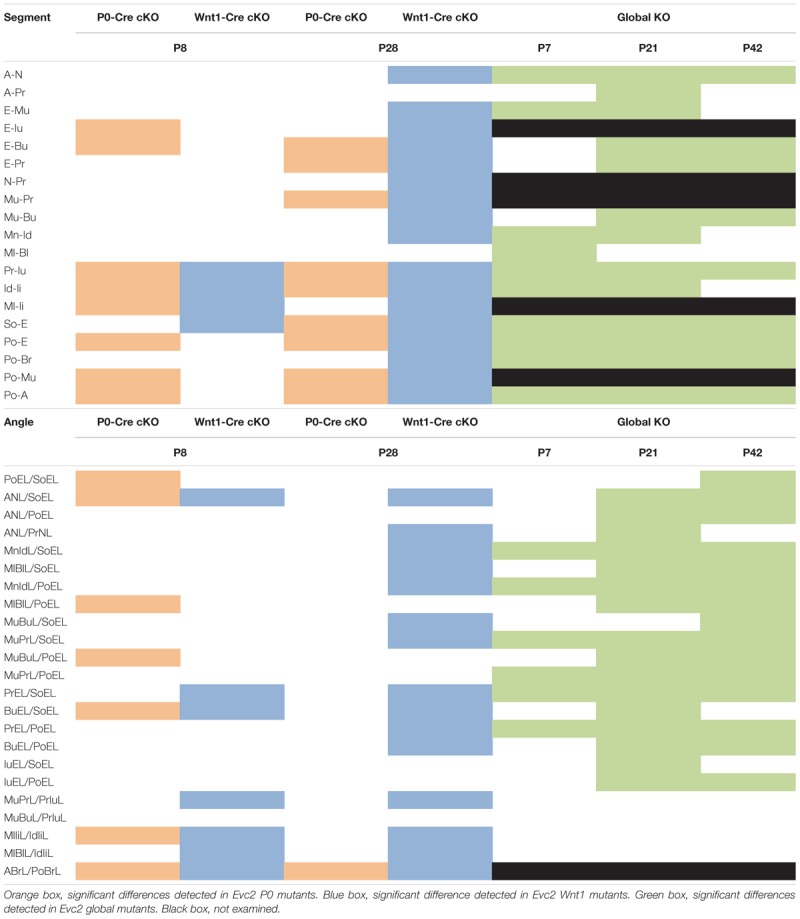

Similar analyses of the *Evc2 Wnt1* mutants revealed abnormalities limited to the incisors and the skull base. We detected decreases in erupted upper incisor (Pr-Iu), erupted lower incisor (Id-Ii), and distance between the first molar and incisor in the mandible (Ml-Ii), respectively (Figure 2C and Table 2). Similar to *Evc2 P0* mutants, these phenotypic observations recapitulate some characteristics of *Evc2* global mutants (Badri et al., 2016a,b). Additionally, we also found a shortened skull base in the *Evc2 Wnt1* mutants, as evidenced by decreased length in the anterior skull base (So-E) (Figure 2C). Although most of the linear measurements showed no significant differences between control and *Evc2 Wnt1* mutants, we observed a slanted mid-facial region in the *Evc2 Wnt1* mutants, supported by the following decreased angle measures: the nasal bone to the skull base (ANL/SoEL), the maxilla-premaxilla to the skull base (MuBuL/SoEL), the premaxilla to the cranial base (PrEL/SoEL), the premaxilla to the cranial vault (BuEL/PoEL), and the angle of the cranial vault (ABrL/PoBrL) (Figure 2D). In summary (Table 2), at P8 both *Evc2 P0* mutants and *Evc2 Wnt1* mutants showed fewer aspects of mid-facial defects compared to *Evc2* global mutants. Of the two types of conditional mutants, *Evc2 P0* mutants showed more severe and widespread abnormalities compared to *Evc2 Wnt1* mutants at P8 (Table 2).

### *Evc2 Wnt1* Mutants Showed More Aspects of Mid-Facial Defects Than *Evc2 P0* Mutants at P28

**FIGURE 3:**
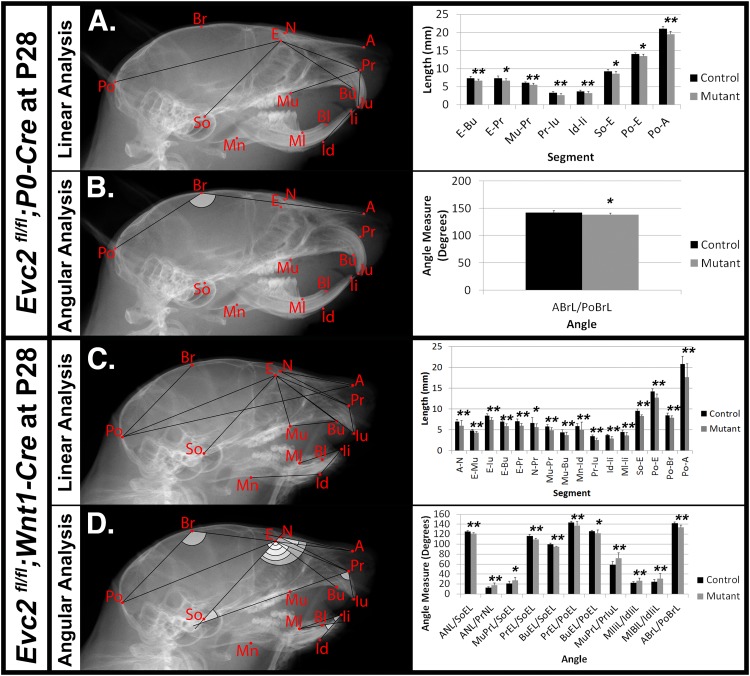
Differential mid-facial defects between *Evc2 P0* mutants and *Evc2 Wnt1* mutants at P28. Lateral X-ray cephalogram indicates the linear **(A)** and angular **(B)** measurements with significant differences between controls and *Evc2 P0* mutants. Lateral X-ray cephalogram indicates the linear **(C)** and angular **(D)** measurements with significant differences between controls and *Evc2 Wnt1* mutants. Graphs indicate the linear and angular values of the measurement with significant differences (*N* = 7; ^∗^*p* < 0.05; and ^∗∗^*p* < 0.01).

Mouse cranial growth rate of peaks and reaches 80% of its adult length within the first month (Vora et al., 2015; Wei et al., 2017). We therefore performed similar lateral cephalometric analyses at P28 but, unlike analyses at P8, observed more widespread craniofacial deficiencies in *Evc2 Wnt1* mutants compared to *Evc2 P0* mutants. In *Evc2 P0* mutants we found significant decreases in: viscerocranial length (E-Bu and E-Pr), distance between molar and incisor in maxilla (Mu-Pr), erupted upper incisor length (Pr-Iu), erupted lower incisor length (Id-Ii), length of anterior skull base (So-E), neurocranial length (Po-E), distance between the first molar to the most posterior point (Po-Mu), and total skull length (Po-A) (Figure 3A). The only significant angle difference was a decreased angle of cranial vault (ABrL/PoBrL) in the *Evc2 P0* mutants (Figure 3B).

In contrast to the *Evc2 P0* mutants, *Evc2 Wnt1* mutants at P28 displayed a more severe and widespread decreases in their craniofacial linear measurements. We found significant decreases in nasal bone length (A-N), nasal bone height (E-Mu), viscerocranial length (E-Bu), and (E-Pr), relative position of pre-maxilla to cranium (N-Pr), distance between molar and incisor in maxilla (Mu-Pr), palatal length (Mu-Bu), length of anterior cranial base (So-E), neurocranial length (Po-E), length of posterior cranium (Po-Br), and total skull length (Po-A). Similar to *Evc2 Wnt1* mutants at P8, we also observed shortened erupted upper (Pr-Iu) and lower (Id-Ii) incisors, and decreased distance between molar and incisor in mandible (Ml-Ii) (Figure 3C). Consistent with the slanted mid-facial region in the gross morphologic observations, we also detected decreased angle measures of nasal bone to cranial base (ANL/SoEL), premaxilla to cranial base (PrEL/SoEL), premaxilla to cranial vault (BuEL/PoEL), and the angle of the cranial vault (ABrL/PoBrL), respectively (Figure 3D). Overall, *Evc2 Wnt1* mutants showed severely affected mid-facial bones and a more slanted mid-facial region at P28 when compared to both control and *Evc2 P0* mutant counterparts.

In Table 2, a series of linear and angular defects detected in *Evc2 P0* mutants and *Evc2 Wnt1* mutants (present study) are compared with those found in *Evc2* global mutants (Badri et al., 2016a,b). At P7/P8, comparisons in linear measurements revealed that all tooth-associated abnormalities detected in *Evc2 P0* mutants and *Evc2 Wnt1* mutants were also detected in the *Evc2* global mutants. Additionally, nasal bone length (A-N), viscerocranial height (E-Mu), and mandibular lingual alveolar bone length (Ml-Bl) were significantly shorter in the *Evc2* global mutants than in controls, whereas these characteristics were normal in both *Evc2 Wnt1* mutants and *Evc2 P0* mutants. In the angular measurements, we found that lower incisor inclination (MlIiL/IdIiL) and upper incisor inclination (MuPrL/PrIuL) were increased in the *Evc2 P0* mutants and *Evc2 Wnt1* mutants but not in the *Evc2* global mutants. At P21/P28, all linear measurement differences detected in *Evc2 Wnt1* mutants were detected in *Evc2* global mutants. Of these, decreased palatal length (Mu-Bu) and length of posterior cranium (Po-Br) were not detected in the *Evc2 P0* mutants. These results reflect subtle phenotypic dissimilarities between mutants at P28 that are not apparent at P8. In the P28 angular measurements, we found that defective nasal bone to cranial vault (ANL/PoEL), maxilla-premaxilla to cranial base (MuBuL/PoEL), and upper incisor to cranial vault in *Evc2 P0* mutants, but not in the *Evc2 Wnt1* mutants, whereas defective upper incisor inclination (MuPrL/PrIuL) and lower incisor inclination (MlIiL/IdIiL) was observed in the *Evc2 Wnt1* mutants but not in the *Evc2* global mutants. Except for the decreased angle of cranial vault (ABrL/PoBrL), there were no other angular differences detected in the *Evc2 P0* mutants.

### Loss of *Evc2* Expression in Neural Crest-Derived Cells Severely Affects Head Shape and Mid-Facial Bone Shapes

**FIGURE 4:**
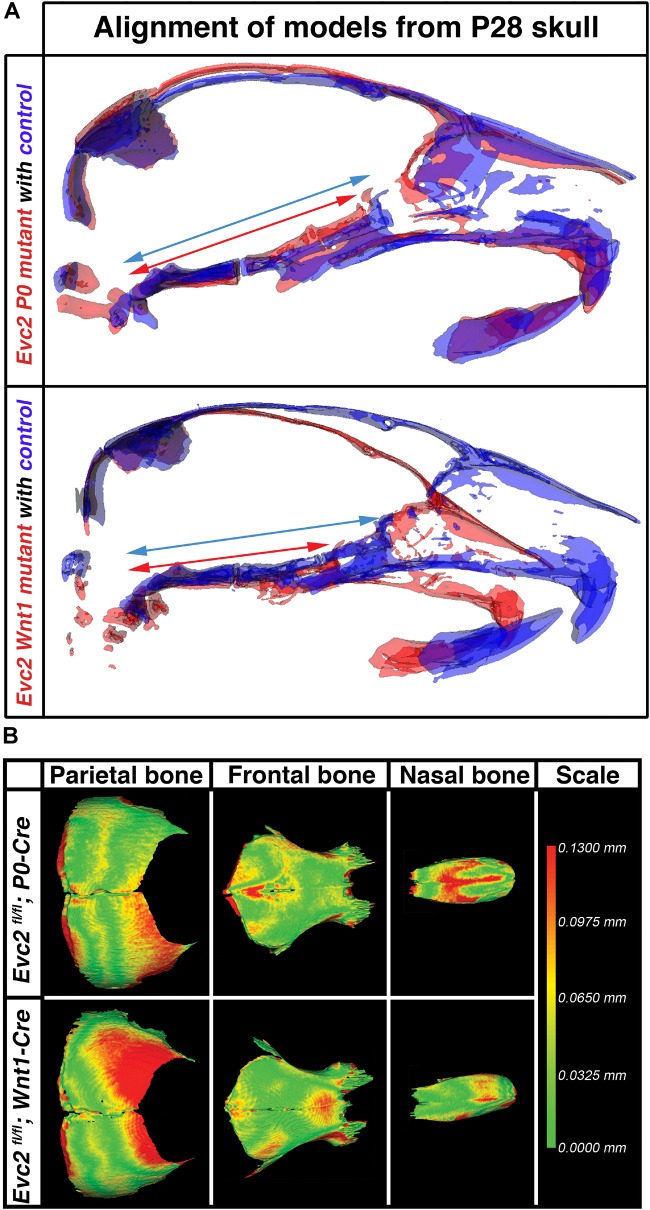
*Evc2* cKO mutants demonstrate skull shape and mid-facial shape differences. **(A)** Mid-sagittal planes were generated based on the micro-CT scans of the controls (Blue), *Evc2 P0* mutants (red) and *Evc2 Wnt1* mutants (red). Models of skulls from mutants and corresponding controls were then superimposed at the occipital bones of the skull. Blue and red arrows are spanning the entire regions of the skull bases in controls and mutants, respectively. **(B)** Nasal, frontal, and parietal bones from P28 *Evc2 P0* mutants and *Evc2 Wnt1* mutants were superimposed with corresponding skull bones from P28 controls. Pictures shown represent comparisons within indicated mutants with corresponding controls. Color indicates the distances the mutants bone surface protruding out of the control surface at the indicated region.

Particularly at P28, *Evc2 P0* mutants showed very limited angular defects. To better appreciate the impacts of *Evc2* mutation on the overall skull shape at P28, we: (1) generated 3D surface models from micro-CT data for the midline portion of the controls, *Evc2 P0* mutants, and *Evc2 Wnt1* mutants at P28 and (2) registered the skulls at the most posterior region (Figure 4A). Registration of each pair of skulls revealed nearly no shape differences in the interparietal and basioccipital bones (i.e., tissues negative for neither *P0-Cre* nor *Wnt1-Cre* expression). When we individually superimposed nasal, frontal, and parietal, respectively, bones, respectively, using *Evc2 P0* mutants and *Evc2 Wnt1* mutants with corresponding controls, we did not see noticeable size differences (Figure 4B). This suggests that there was no overt overall growth retardation since general growth retardation would affect all bones. Morphometric comparison, however, revealed mutant-specific structural differences. *Evc2 Wnt1* mutants had a more shortened and downward-angled facial region than *Evc2 P0* mutants had (Figure 4A). Since the skull base connects the facial region with the posterior part of the skull, the extent of skull base linear differences may directly result in angular measurement differences in *Evc2 Wnt1* and *Evc2 P0* mutants, respectively. To visualize the potential shape or scaling differences within each individual bone, we digitally extracted nasal, frontal, and parietal bones separately and superimposed control surface models with corresponding mutant samples (Figure 4B). Superimpositions of individual bones revealed shape differences in all nasal, frontal, and parietal bones (Figure 4B). Most strikingly, in the parietal bones (non-neural crest derived), we detected deviations in the anterior regions, in which the mutant surfaces “stuck out” of the control surfaces (red color in Figure 4B). *Evc2 Wnt1* mutants have more severely affected parietal bones than *Evc2 P0* mutants. Three other mutant skulls from each mutant group showed similar patterns when compared with littermate controls (Supplementary Figure S1). When we compared four littermate controls within the *Evc2 P0* mutant group or four littermate controls within the *Evc2 Wnt1* mutant group using the same standard, almost all areas showed green color indicating that surface structures of those bones are highly consistent among controls (Supplementary Figure S1, one pair of comparison for each group is shown). The defective skull bones in *Evc2* mutants likely reflect the shortened skull base and resultant shortened and downward-angled mid-facial region. Therefore, the more severely affected parietal bones in the *Evc2 Wnt1* mutants (compared to *Evc2 P0* mutants) are likely due to the more severely affected skull base in these mutants, as previously discussed (Table 2).

### The Differential Early Fusion of the Presphenoidal Synchondrosis Leads to the Differentially Shortened Skull Base in the *Evc2 P0* and *Evc2 Wnt1* Mutants

**FIGURE 5:**
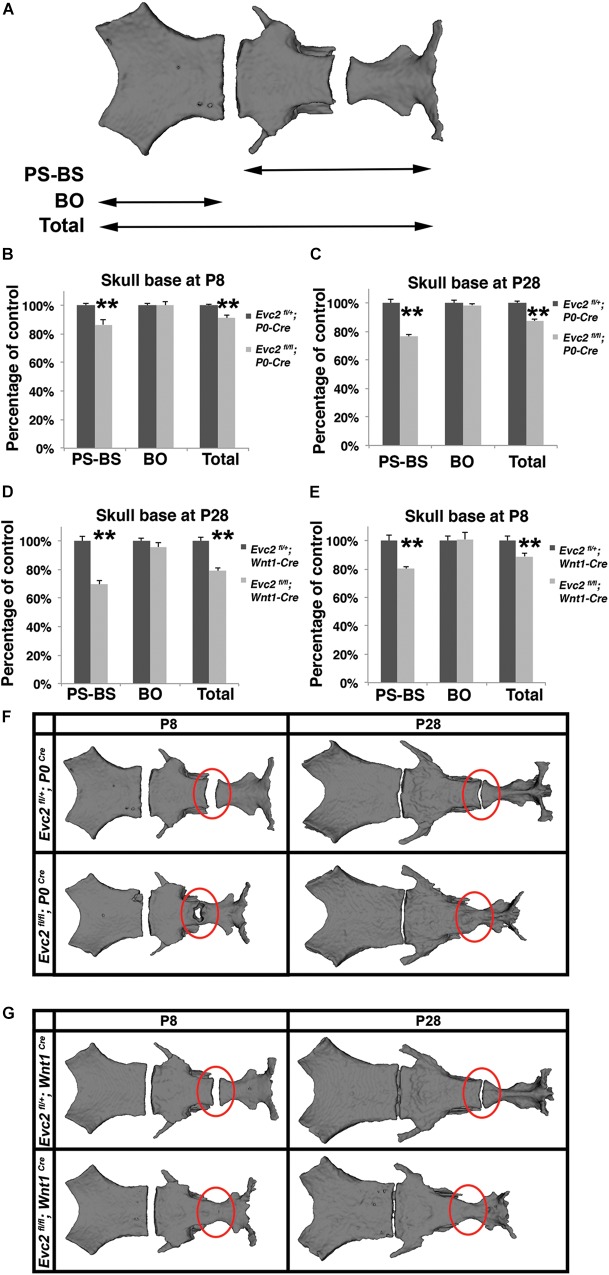
*Evc2* mutants have defective skull bases. **(A)** Diagram of measurements in labels **(B–E)**. Quantification of skull base length at P8 **(B,D)** and P28 **(C,E)**. The length of the skull base was determined by assessing the linear distance shown in label **(A)** (*N* = 5; ^∗∗^*p* < 0.01 comparing to controls). Surface models of skull bases were generated based on micro-CT scans of P28 **(F)** and P8 samples **(G)**. Red circles indicating the ISS or fused ISS.

The skull base is a unique structure that connects multiple bones in the skull and has a significant influence on facial growth, particularly in the mid-facial region (Nie, 2005). Our studies demonstrated that *Evc2* is expressed in both intersphenoidal synchondrosis (ISS) and the spheno-occipital synchondrosis (SOS) (Supplementary Figure S2). Quantification of the skull base length (described in Figure 5A) revealed that, compared to controls, skull bases from *Evc2 Wnt1* mutants are 20% shorter while skull bases from *Evc2 P0* mutants are only 10% shorter at P28 (Figures 5B–E, *N* = 5, *p* < 0.01). Elongation of the skull base is supported by the endochondral ossification within the ISS and the SOS. To investigate whether the abnormal ISS or SOS is the cause of the affected skull base elongation in either *Evc2 P0* or *Evc2 Wnt1* mutants, we generated surface models from the skull bases of *Evc2 P0* and *Evc2 Wnt1* mutants, respectively. At P28, we observed premature fusion of ISS in both *Evc2 P0* and *Evc2 Wnt1* mutants (Figures 5B,C). However, at P8, we observed premature fusion of ISS in *Evc2 Wnt1* mutants and partial fusion of ISS in the *Evc2 P0* mutants (Figures 5B,C). These results suggest that the premature fusion of the ISS in *Evc2 P0* mutants happens between P8 and P28, while the premature fusion of ISS in *Evc2 Wnt1* happens before P8 (Figure 5C).

**FIGURE 6:**
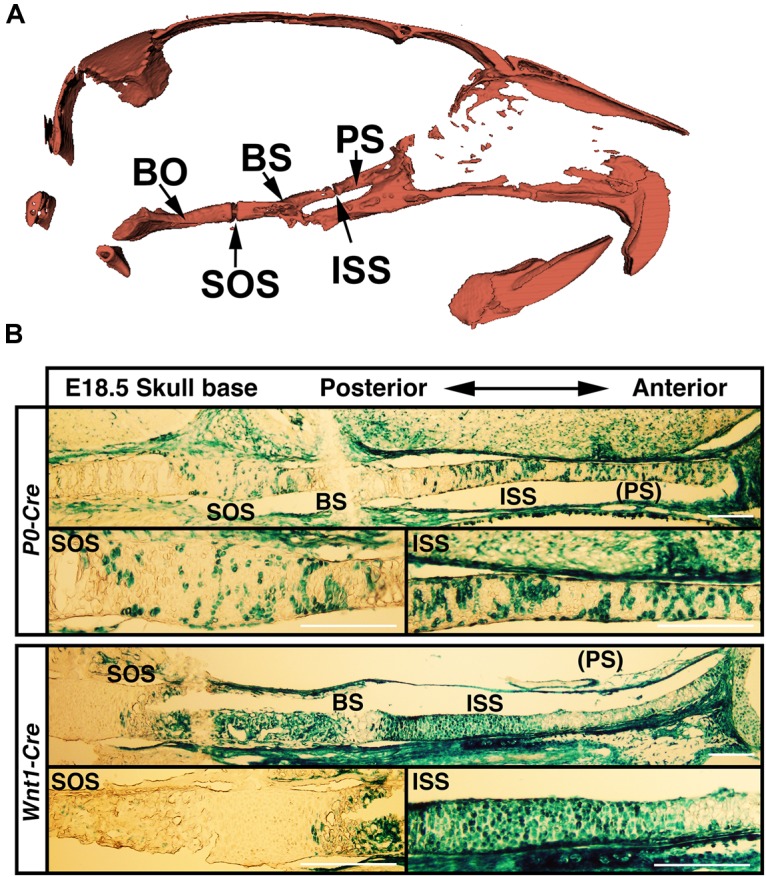
**(A)** Diagram to skull base structure. **(B)** Differential Cre-dependent recombination in the skull bases of *P0* and *Wnt1* Cre lines. Indicated Cre mice were crossed with Rosa26-LacZ Cre reporter mice, followed by beta-galactosidase activity staining to visualize the Cre recombinant cells in the skull base. PS, presphenoid bone; ISS, intersphenoidal synchondrosis; BS, basisphenoid bone; SOS, spheno-occipital synchondrosis. Scale bar = 100 μm.

The anterior regions of the skull base are derived from neural crest cells and are the active sites for postnatal skull base elongation (Nie, 2005). To examine if differential skull base lengths in *Evc2 P0* mutants and *Evc2 Wnt1* mutants result from differential Cre recombination efficiency in the anterior part of the skull base, we examined the Cre recombined cells in the anterior region of the skull base. At E18.5, while *P0-Cre* leads to sporadic recombination within the chondrocytes in the anterior part of the skull base, *Wnt1-Cre* leads to nearly 100% recombination in the chondrocytes in the same region (Figure 6). These results suggest that (1) *Evc2* function within the ISS is critical for maintaining the ISS and supporting the elongation of skull base; and that (2) differential *Evc2* deletion efficiency mediated by *P0-Cre* and *Wnt1-Cre* leads to differential impacts upon skull base elongation, which secondarily affects differential outcomes in mid-facial abnormalities between *P0-Cre* and *Wnt1-Cre* mutants.

## Discussion

Craniofacial abnormalities have been reported in EvC patients (Ellis and van Creveld, 1940; McKusick et al., 1964; Eidelman et al., 1965; Prabhu et al., 1978). Despite our recent studies describing mouse models that recapitulate the craniofacial abnormalities of EvC patients (Badri et al., 2016b), the underlying pathophysiological mechanisms of these abnormalities remain largely unknown. In this study, we deleted *Evc2* in the neural crest derived cells using *P0-Cre* and *Wnt1-Cre*. While both of these Cre lines equally target the mid-facial region, they differentially target the anterior region of the skull base. Comprehensive analysis of these two types of *Evc2* mutant lines allows us to delineate the craniofacial abnormalities due to *Evc2* loss of function in the neural crest derived tissues and the secondary abnormalities due to the affected skull base. The results here are potentially applicable to understand postnatal mid-facial development in other types of ciliopathies.

Compared to their global mutant counterparts, we believe that two types of *Evc2* cKO mice, *Evc2 P0* mutants and *Evc2 Wnt1* mutants, are great tools in helping to elucidate the pathological mechanism leading to the mid-facial defects. Our phenotypic analysis at P8 and P28 indicated that mid-facial defects were recapitulated in *Evc2 Wnt1* mutants, but to a less extent in *Evc2 P0* mutants. The *Evc2 P0* mutants show a smaller head (Figure 1A, 2nd row) and differences in some of the parameters (Figures 2A,B) at P8, which are consistent with our previous report (Kwon et al., 2018). However, many of the differences are compensated by P28 (Figure 1B, second row and Figures 3A,B). In contrast, the *Evc2 Wnt1* mutants show comparable head size at P8 but become smaller at P28 (Figures 1A,B, fourth row), and more parameters show differences at P28 than P8 (Figures 2C,D, 3C,D). Those facts strongly suggest that tissues that are specifically positive for *Wnt1*-Cre mediated recombination play a critical role for post-natal mid-facial development. Considering the impacts of shortened skull base on the mid-facial region as shown in Figure 4A and that loss of *Evc2* leads to early fusion of ISS in *Evc2 Wnt1* mutants, we believe that the differential *Evc2* deletion efficiency mediated by *P0-Cre* and *Wnt1-Cre* at the anterior region of the skull base is a major reason leading to the differential mid-facial defects in these two types of *Evc2* cKO mice.

Differential phenotypic differences between *Evc2* global mutants, *Evc2 P0* mutants and *Evc2 Wnt1* mutants suggest that different pathological mechanisms lead to various craniofacial defects due to *Evc2* mutations. For example, viscerocranial length (E-Bu and E-Pr), erupted upper incisor length (Pr-Iu), erupted lower incisor length (Id-Ii), cranial base length (So-E) are due to the *Evc2* loss of function within the neural crest cells. In contrast, the nasal bone to cranial base (ANL/SoEL), nasal bone to premaxilla (ANL/PrNL), premaxilla to cranial base (PrEL/SoEL), upper incisors to cranial base (BuEL/SoEL), premaxilla to cranial vault (PrEL/PoEL), premaxilla to cranial vault (BuEL/PoEL), upper incisor inclination (MuPrL/PrIuL), lower incisor inclination and (MlIiL/IdIiL), are due to the unique spatial activity of the *Wnt1-Cre* driver. Since the skull base connects the posterior and mid-facial regions, the shortened skull base is a well-known cause of mid-facial defects (Nie, 2005; Vora et al., 2015). Therefore, our data suggest that the differential Cre recombination efficiency mediated by the *Wnt1-Cre* and *P0-Cre* lineage cells within the anterior part of the skull base leads to the differential impacts on the pre-mature fusion of the ISS, which connects the presphenoid bone and the basisphenoid bone, and thus the length of the skull base. Those structural changes secondarily lead to the shortened and downward angled facial defects observed more prominently in the *Evc2 Wnt1* mutants.

Similar to the appendicular bones, the skull base develops through endochondral ossification, wherein the cartilage primordium forms first, followed by mineral deposition. The proliferation and maturation of chondrocytes at the synchondrosis in the skull base is quite important for skull base elongation (Nie, 2005). In this study, we found loss of function of *Evc2* results in premature closure of the ISS before P8 in *Evc2* global mutants and in *Evc2 Wnt1* mutants. We previously reported that loss of function of *Evc2* results in severe chondrodysplastic dwarfism; however, we never observed premature closure of appendicular growth plates through adult stages (Zhang et al., 2016a). Those data suggest that a function of *Evc2* to maintain cartilage is different between appendicular growth plates and synchondroses. A potential mechanism leading to the premature fusion of the ISS is a subject under active investigation.

In this study, we used both *P0-Cre* and *Wnt1-Cre* to specifically delete *Evc2* in the neural crest derived cells. Despite largely overlapping targeted regions within the facial regions of *P0-Cre* and *Wnt1-Cre* (Danielian et al., 1998; Yamauchi et al., 1999) (our own comparisons, data not shown), small differences have been noticed between those Cre lines by multiple studies (Wang et al., 2011; Liu et al., 2012; Suzuki et al., 2013; Chen et al., 2017). Particularly, our recent studies demonstrated that both *P0-Cre* and *Wnt1-Cre* differentially target neural crest cells emerging at the anterior, middle and posterior portions of the head during embryogenesis (Chen et al., 2017). Previously, we reported a neural crest-specific disruption of TGF-beta activating kinase 1 (*Tak1*) using *Wnt1*-*Cre* and *P0*-*Cre*, respectively (Yumoto et al., 2013; Liu et al., 2018). While *Tak1 P0* mutants do not show overt craniofacial phenotypes at birth (Liu et al., 2018), *Tak1 Wnt1* mutants develop cleft palate secondarily to the micrognathia (Yumoto et al., 2013). This is another example that *Wnt1*-*Cre* and *P0*-*Cre* result in distinctive phenotypes. In the current study, we identified that *P0-Cre* and *Wnt1-Cre* differentially target the anterior regions of the skull base, and we took advantage of those differences to identify the pathophysiological mechanism leading to the mid-facial defects in *Evc2* mutant mice. However, there is a formal possibility that the differential Cre activity within the unknown types of cells or tissues contributing to the differential mid-facial defects in *Evc2 P0* mutants and *Wnt1* mutants. We will further investigate an involvement of the premature fusion of the ISS to the mid-facial defects in our mutant mouse models.

*Wnt1-Cre* is known to target the brain (Danielian et al., 1998), while *P0*-Cre is not expressed in the central nervous system. However, it is unlikely that the differential mid-facial defects observed in the *Evc2 Wnt1* mutants and *Evc2 P0* mutants are secondary effects of the differential Cre targeting within the brain. Firstly, the mid-facial region is not in direct contact with the brain and thus brain development should not have an impact on the development of the mid-facial region. Secondly, despite the fact that *Evc2* is a positive regulator of Hedgehog signaling, there are no brain developmental defects observed in patients or mice. Recent studies also suggest that the transgenic construct in *Wnt1-Cre* mouse line transiently elevates WNT signaling during craniofacial development (Lewis et al., 2013). It is unlikely that this is the reason leading to differential mid-facial defects observed in the *Evc2 Wnt1* mutants and *Evc2 P0* mutants. Our analysis demonstrated that no overt head shape differences between *Evc2^fl/+^* and *Evc2^fl/+^; Wnt1-Cre*, suggesting the transiently elevated WNT signaling due to *Wnt1*-*Cre* did not lead to craniofacial abnormalities.

By specifically deleting *Evc2* in neural crest-derived tissues, we were able to generate a mouse model of human EvC that recapitulates the mid-facial defects associated with EvC syndrome. Multiple analyses at different developmental stages confirm the critical role of the skull base during face elongation and development within the first month and demonstrate that the mid-facial defects in EvC syndrome are largely due to the premature fusion of the ISS in the skull base. Examination and targeting the shortened skull is therefore a suggested therapeutic option for the mid-facial defect in EvC patients.

## Author Contributions

AK, KL, YMi, and HZ conceived this study. AK, KL, and HZ performed the analysis. AK, KL, MY, ACOR, YMo, LC, YMi, and HZ contributed to data interpretation and critical revision of the manuscript. AK, KL, YMi, and HZ drafted the manuscript.

## Conflict of Interest Statement

The authors declare that the research was conducted in the absence of any commercial or financial relationships that could be construed as a potential conflict of interest.

## Footnotes

1 www.itksnap.org

2 www.slicer.org

## Supplementary Material

FIGURE S1 Superimposition of each skull bones within the *Evc2 P0* group. Comparisons of two controls (*Evc2 P0* C vs. C) and three additional pairs of control and mutant (*Evc2 P0* C vs. m) are shown. **(B)** Superimposition of each skull bones within the *Evc2 Wnt1* group. Comparisons of two controls (*Evc2 Wnt1* C vs. C) and three additional pairs of control and mutant (*Evc2 Wnt1* C vs. m) are shown.

FIGURE S2 *Evc2* expression in synchondrosis in the skull base. Beta-galactosidase activity from the *lacZ* knock-in allele of *Evc2* locus was used as a surrogate of the expression of *Evc2*. The skull bases from *Evc2^lacZ/+^* at P10 were stained with X-gal followed by eosin counter staining. Robust staining (blue) was detected in the ISS and the SOS. *Evc2^+/+^* littermates were used as negative controls.

TABLE S1 Lateral X-ray cephalogram indicates the linear and angular measurements within P0-Cre group at P8 and P28 and within Wnt1-Cre group at P8 and P28. Within each group, the potential gender specific differences were evaluated by comparing control male with control females; and mutant males and mutant females. Occasional significant differences observed were marked in yellow color.
