# Supplementary figures and images for: A Ciliary Protein EVC2/LIMBIN Plays a Critical Role in the Skull Base for Mid-Facial Development
Source: Front Physiol. 2018 Oct 25;9:1484. doi: 10.3389/fphys.2018.01484 (PMC6210651; doi:10.3389/fphys.2018.01484)

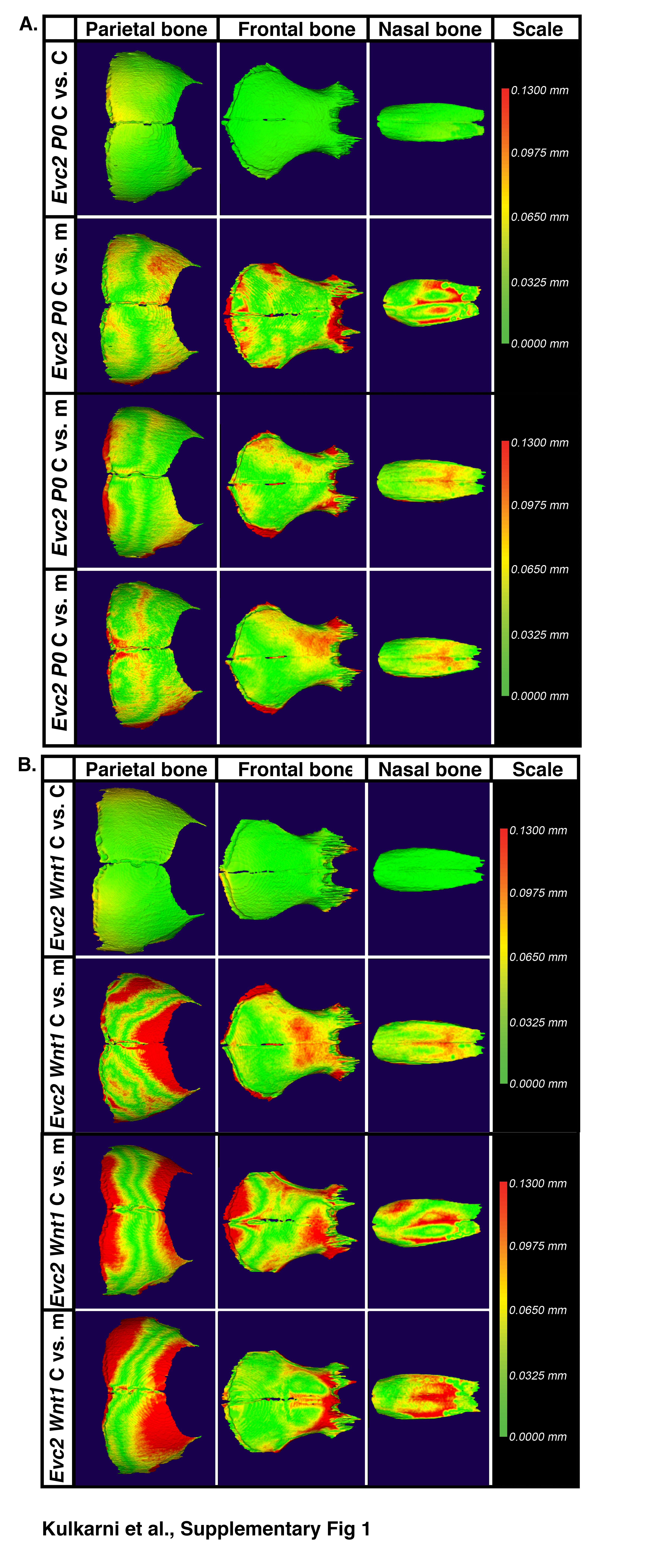

Supplement: FIGURE S1 — Superimposition of each skull bones within the Evc2 P0 group. Comparisons of two controls (Evc2 P0 C vs. C) and three additional pairs of control and mutant (Evc2 P0 C vs. m) are shown. (B) Superimposition of each skull bones within the Evc2 Wnt1 group. Comparisons of two controls (Evc2 Wnt1 C vs. C) and three additional pairs of control and mutant (Evc2 Wnt1 C vs. m) are shown. [file Image_1.TIF]

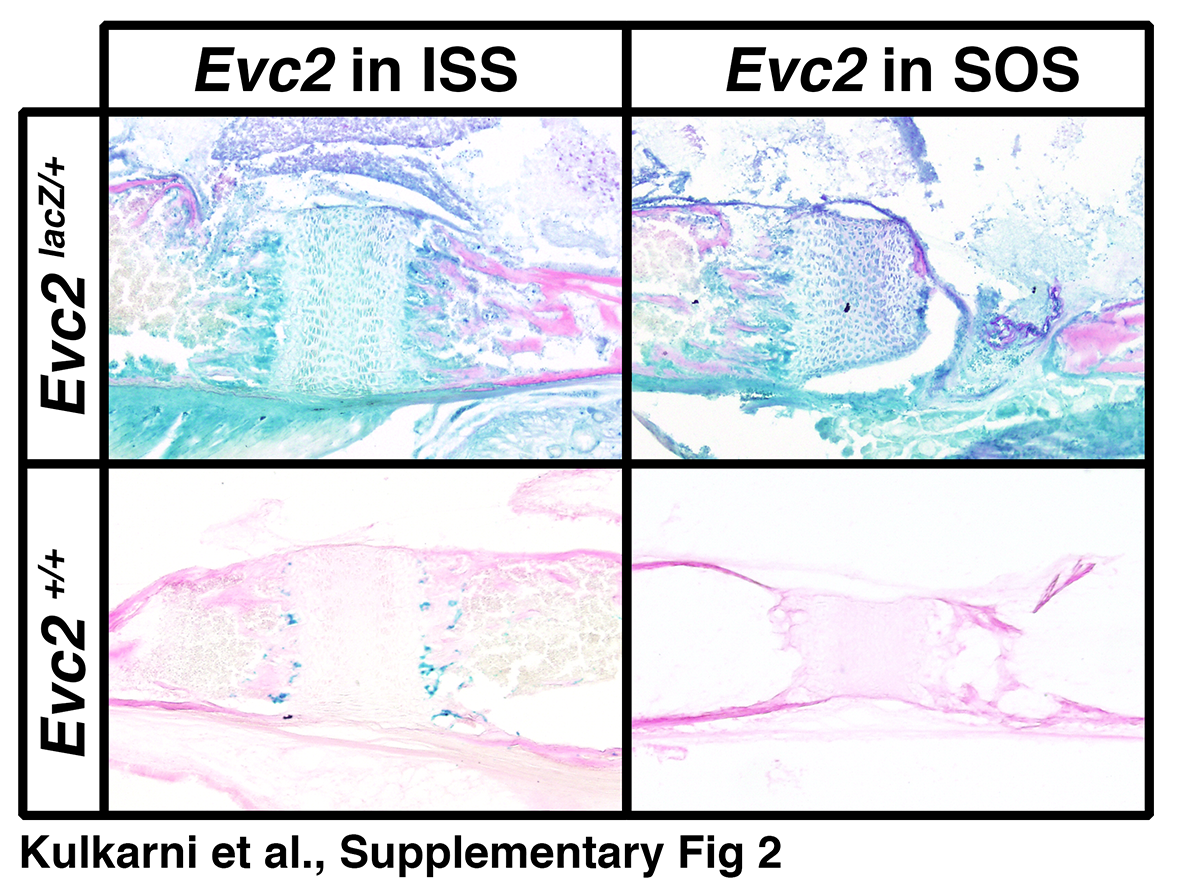

Supplement: FIGURE S2 — Evc2 expression in synchondrosis in the skull base. Beta-galactosidase activity from the lacZ knock-in allele of Evc2 locus was used as a surrogate of the expression of Evc2. The skull bases from Evc2lacZ/+ at P10 were stained with X-gal followed by eosin counter staining. Robust staining (blue) was detected in the ISS and the SOS. Evc2+/+ littermates were used as negative controls. [file Image_2.TIF]
